# Litter eco-hydrological function characteristics of three typical plant communities in the area of Karst peak-cluster depressions from Guizhou, China

**DOI:** 10.1371/journal.pone.0278565

**Published:** 2022-12-06

**Authors:** Jianli Zhang, Ting Zhang, Lihua Pu, Lingbin Yan, Guojun Cai, Pengli Chen, Tao Yang, Chen Zhang

**Affiliations:** 1 College of Eco-environmental Engineering, Guizhou Minzu University, Guiyang, Guizhou, China; 2 The Karst Environmental Geological Hazard Prevention of Key Laboratory of State Ethnic Affairs Commission, Guizhou Minzu University, Guiyang, Guizhou, China; 3 College of Biological Sciences, Guizhou University, Guiyang, Guizhou, China; 4 Institute of Mountain Resources of Guizhou Province, Guiyang, Guizhou, China; Georgia Southern University, UNITED STATES

## Abstract

Litter is an important component of forest ecosystems and plays an important eco-hydrological function. Many studies have been carried out on litter at present, but less research has been carried out on the eco-hydrological service functions of litter in different plant communities in Karst, especially in the area of Karst peak-cluster depressions in southwest China. To reveal the characteristics of the hydrological function of the litter layer of the plant community in the area of Karst peak-cluster depressions around FAST (Five-hundred-meter Aperture Spherical Radio Telescope), three typical plant community litter layers of the broad-leaved forest, coniferous forest, and shrub were selected as research objects, and the hydrological function of the litter layer of different plant community types was studied using the immersion method. The results indicated: 1) The litter layer of the broad-leaved forest plant community has the strongest function of intercepting and regulating precipitation (*M*_*lmax*_ = 24.17±0.33 t/ha, *M*_*sv*_ = 19.93±0.21 t/ha), and its hydrological service function is the best. 2) The higher the decomposition degree of litter, the stronger the interception function. 3) The fitted equations for both the litter water-absorption capacity (*Q*_*ct*_) and time (*t*) for plant communities were *Q*_*ct*_ *= b + a*ln*t*, and the fitted equations for both the litter water-release capacity (*Q*_*st*_) and time (*t*) were *Q*_*st*_ *= a t* ^*b*^. 4) The fitted equations for both the water absorption and release rates (*v*_*c*_ and *v*_*s*_) and time (*t*) of the litter were *v = a t* ^*-b*^. The water absorption rates of litter were the fastest within 5 min (15529.01~22634.43 g/kg·h), with the greatest interception and storage function for short-term rainfall.

## 1 Introduction

Forest ecosystems are important terrestrial ecosystems that play a huge regulating role in the atmospheric water cycle. Forest ecosystems have ecological service functions, such as soil and water conservation, water purification, and climate regulation [1]. As the main body of the forest ecosystem, plant communities play hydrological service functions through the plant canopy, litter layer, and soil layer. Furthermore, litter is an important intermediate link between vegetation and soil material circulation and energy flow [2, 3]. In forests, litter is an important water storage container [4]. Litter has an obvious capacity for interception and storage and can intercept precipitation, reduce soil erosion, delay surface runoff, and improve the structure and composition of the soil, thereby playing a role in ecological hydrology [5, 6]. The hydrological function of litter is closely related to the composition and structure of the plant community as well as the litter input, litter reserves, decomposition degree, temporal and spatial distribution pattern, and water-holding capacity. Generally, the more the litter reserves, the thicker the litter layer and the higher the decomposition degree, and the better its hydrological regulation ability. Along with the decomposition of litter, its structure also changes, which can translate into water-holding capacity and water-absorption [7].

Scholars have carried out a large number of studies on the ecological functions of forest litter in China and other countries, mainly focusing on the litter reserves [8, 9], hydrological function [10], decomposition process and rate [11–13], nutrient release characteristics [14, 15], ecological stoichiometry [16, 17], characteristics of the microbial community in the litter layer [18, 19], the response of litter decomposition to the environment [20, 21], and decomposition law model [22]. Litter is abundant in Pacific Northwest forests that have been without fire and timber harvest long enough for trees to grow, die, and fall [23]. In a study of changes in the physical properties and water repellency of litter in central Poland, litter was found to increase water storage in forest ecosystems [24]. Paletto and Tosi [25] note the physical feature of the wettability of litter is closely related to water storage capacity and absorbability in the Mediterranean climate. In the study by Soichiro and Koichi [26], climate change may alter the balance between litter and aboveground biomass by changing aboveground net primary production and decomposition rates along elevation gradients in central Japan. With the deepening of research on litter ecology, an increasing number of experts and scholars in the fields of ecology, forestry, and environmental science have focused on litter function [8, 27–29].

In the Karst region of southwest China, the expansion of stone desertification has led to a dramatic decline in woodland cover and a high degree of spatial heterogeneity in the distribution of nutrients in the litter. Karst landscapes are widely distributed in Guizhou Province, with a variety of landform types. Karst forest habitats have characteristics that are distinctly different from other forest habitats, such as high rock exposure, multi-layered ecological space, high heterogeneity of habitats, shallow soils, and low water-holding capacity of soils. At present, research on the hydrological functions of litter has mainly focused on the effects of different vegetation types [1, 30], site conditions [31, 32], and disturbance and intensity [33] on litter reserves and hydrological functions, but there are few reports on the litter reserves and eco-hydrological function in the area of Karst, especially in the area of Karst peak-cluster depressions. FAST is the world’s largest astronomical telescope currently, and the surrounding area is typical Karst peak-cluster depressions. With the increasing Karst desertification, and with FAST running many astronomical observation missions, it is particularly important to protect the surrounding ecology. Therefore, it is important to study the eco-hydrological functions of the litter layer of typical plant communities around FAST for soil and water conservation and water resources management in Karst peak-cluster depressions and even in forests around the world.

The aims of this study were to clarify plant communities in the area of Karst peak-cluster depressions: (1) What is the water-holding capacity and reserves of the litter layer? (2) What are the differences between the hydrological functions of the litter layers (the maximum interception capacity, the maximum interception rate, the effective interception capacity, and the effective interception rate)? (3) What are the processes and rates of water absorption and release in the litter layers?

## 2 Research methods

### 2.1 Study area and litter sample collection

The study area is located in southern Guizhou Province, in western Pingtang County, Qiannan Buyi, and Miao Autonomous Prefecture (106°50’32″–107°11’47″ E; 25°38’19″–25°59’09″ N) [34]. The study area belongs to the transition zone from the southeast slope of Yunnan Guizhou Plateau to the hills of Guangxi, 15 km away from Kedu town. It belongs to the typical peak-cluster depressions landform type, with typical rocky desertification, complete Karst development, and concentrated peak-cluster depressions. The soil consists of lime soil and yellow soil [35]. This area belongs to the mid-subtropical monsoon humid climate zone, with an average annual temperature of 17°C, an extremely low temperature of −3.5°C, annual precipitation of 1259 mm/year, a frost-free period of 312 days, and 1637 hours of annual sunshine [34, 36]. The vegetation type belongs to the sub-zone of the subtropical evergreen broad-leaved forest, mainly including evergreen broad-leaved forest, evergreen and deciduous mixed forest, coniferous, and broad-leaved mixed forest, and most bamboo forest or bamboo-wood mixed forest. The plant community is mainly composed of *Fagaceae*, *Magnoliaceae*, *Hamameliaceae*, and *Oleaceae*, with rich plant species [37].

**Fig 1:**
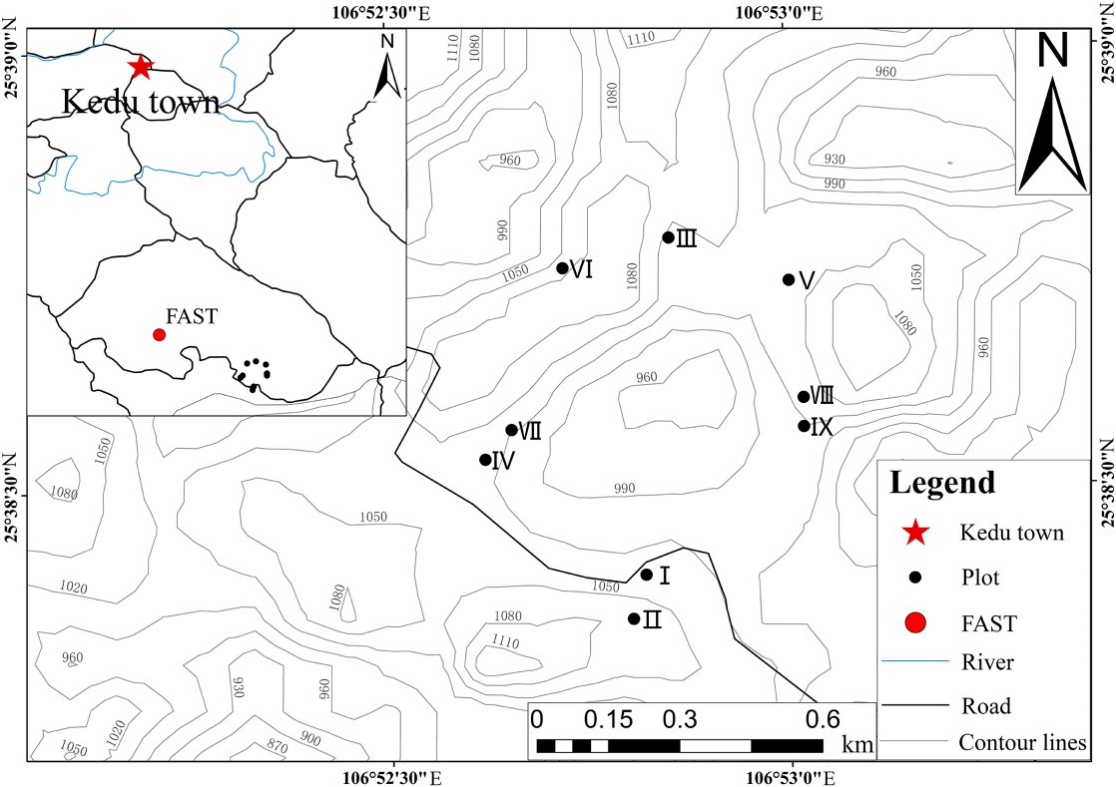
Location of sampling area.

In mid-July 2015, based on a survey of plant communities around FAST, three typical plant community types, including shrubs, broad-leaved forest, and coniferous forest, were selected for the research objects. Sampling surveys were carried out in weather conditions with no rainfall for a week. The study area contained each typical type of plant distributed in the region. Three litter sample collection areas were set up in each of the three community types, for a total of nine litter collection areas. I, II and III are shrub communities, IV, V and VI are broad-leaved forest communities, and VII, VIII and IX are coniferous forest communities (shown in Fig 1). In each litter collection area, three 100 cm × 100 cm litter collection plots were randomly set up, according to the undecomposed layer, the semi-decomposition layer, and the decomposition layer. These levels were sampled in order to collect the litter in each area thoroughly [38].

### 2.2 Determination of litter reserves and moisture content characteristics

The litter samples of different types of plant communities were returned to the laboratory. The soil, rocks, and roots were removed and weighed. The samples were then placed in an oven at 80° C to dry them to constant weight, and the wet weight and dry weight of the samples were recorded. The formulas for the reserves of the litter and the moisture content of the litter are as follows [39]:

$$M=M_{d}/100$$

$$R_{n}=(M_{w}-M_{d})/M_{d}\times 100\%$$

Where *M* is the reserves of the litter (t/ ha), *M*_*d*_ is the dry weight of the litter (g/m^2^), *M*_*w*_ is the wet weight of the litter (g/m^2^), and *R*_*n*_ is the moisture content of the litter (%).

### 2.3 Determination of the ecological and hydrological functions of the litter layer

The litter samples of different types of plant communities were dried in an oven at 80°C to a constant weight, placed in a mesh bag with a hole diameter of 1 mm × 1 mm, and soaked in clean water for 48 h. When the litter net bag was no longer dripping, it was weighed to obtain its wet weight. The actual water-holding rate of litter is approximately 85% of the maximum water-holding rate, and the maximum water-holding rate is generally multiplied by a factor of 0.85 to estimate the effective interception capacity of litter [40]. The formulas for the maximum water-holding capacity, the maximum water-holding rate, the maximum interception capacity, the maximum interception rate, the effective interception amount, and the effective interception rate of the litter layer are as follows [3, 41]:

$$R_{max}=(M_{48w}-M_{d})/M_{d}\times 100$$

$$M_{wmax}=R_{max}\times M$$

$${Rl}_{max}=R_{max}-R_{n}$$

$$M_{lmax}={Rl}_{max}\times M$$

$$R_{sv}=0.85R_{max}-R_{n}$$

$$M_{sv}=R_{sv}\times M$$

Where *R*_*max*_ is the maximum water-holding rate of litter (%), *M*_*48w*_ is the wet weight of the litter after 48 hours of soaking in water (g), *M*_*wmax*_ is the maximum water-holding capacity of the litter (t/ha), *Rl*_*max*_ is the maximum interception rate of litter (%), *M*_*lmax*_ is the maximum interception capacity of litter (t/ha), *R*_*sv*_ is the effective interception rate of litter (%),0.85 is the effective interception coefficient of litter, and *M*_*sv*_ is the effective interception capacity of litter (t/ha).

### 2.4 Determination of “absorption-release” water characteristics of the litter layer

Put the litter of different types of plant communities after drying into a 1 mm ×1 mm aperture nylon mesh bag in an 80℃ oven, tie the mouth of the bag and weigh it, and then soak it in water for 5 min, 15 min, 30 min, 1 h, 2 h, 4 h, 8 h, 12 h, 16 h, 24 h, 48 h, pick it up, weigh the litter net bag when it is no longer dripping, and use it to analyze the process of litter water absorption. Then the litter was allowed to stand for 5 min, 15 min, 30 min, 1 h, 2 h, 4 h, 8 h, 12 h, 16 h, 24 h, 48 h, and then weighed to analyze the water release process of the litter [3, 42]. The calculation formula for the “absorption- release” water function characteristics of the litter layer is as follows:

$$Q_{ct}=(M_{ct}-M_{d})/M_{d}\times 1000,$$

$$Q_{st}=(M_{48w}-M_{st})/M_{d}\times 1000,$$

$$v_{c}=Q_{ct}/t,$$

$$v_{s}=Q_{st}/t,$$

where *Q*_*ct*_ is the water-absorption capacity of litter during immersion for time t (g/kg), *M*_*ct*_ is the wet weight of litter after immersion for time t (g), *M*_*d*_ is the dry weight of litter (g), *Q*_*st*_ is the capacity of water-released by litter during time t (g/kg), *M*_*48w*_ is the wet weight of the litter after 48 hours of soaking in water (g), *M*_*st*_ is the wet weight of litter after standing for time t (g), *v*_*c*_ is the water absorption rate of litter (g/kg*·*h), *v*_*s*_ is the water release rate of litter (g/kg*·*h), and *t* is the litter immersion (standstill) time (h).

### 2.5 Analysis methodology

In the process of conducting the litter statistical analysis of typical plant communities, Microsoft Office Excel 2016 software was used to sort out data, IBM SPSS Statistics 25 software was used for one-way ANOVA, correlation analysis, and regression analysis, and the LSD was used for post hoc multiple comparisons. Sigma plot 14.0 software was used for drawing figures.

## 3 Results and analysis

### 3.1 Analysis of litter layer reserves, water moisture content, and maximum water-holding capacity

**Fig 2:**
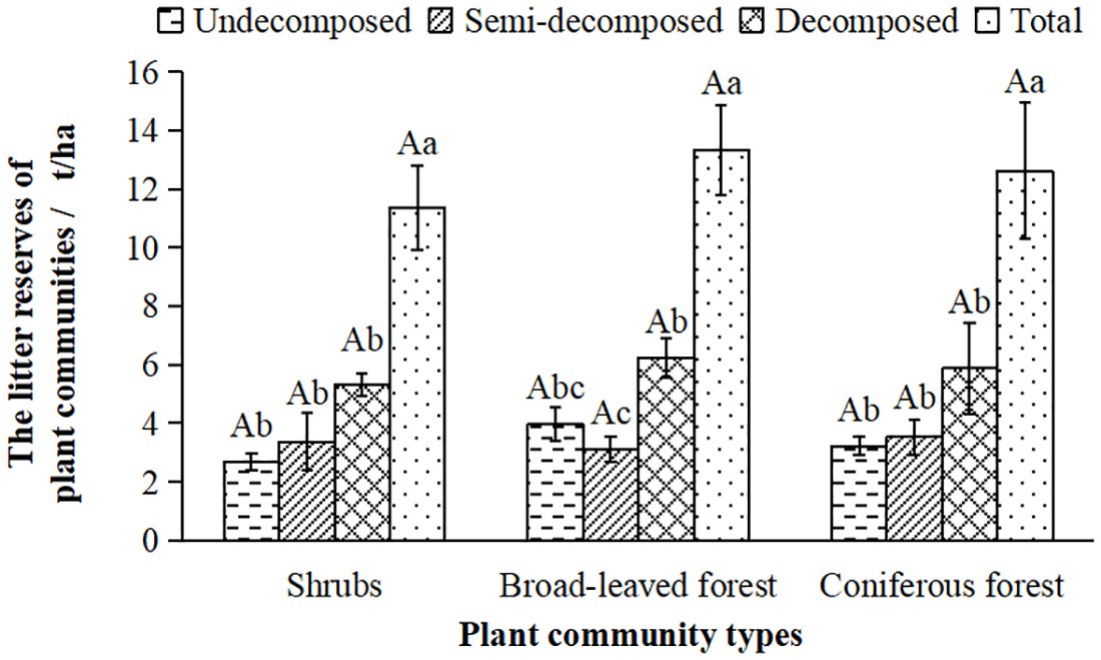
The litter reserves (*M*) in the typical plant communities. Note: Lowercase letters indicate within-group differences and uppercase letters indicate between-group differences (ANOVA followed by LSD, *P*<0.05). The same is below.

The litter layer is an important component of the hydrological function of a plant community. Its reserves directly affect the ecological and hydrological functions of the plant community. As shown in Fig 2, there was no significant difference in the total litter reserves(*M*) of the three typical plant communities, and the trend of the total *M* was broad-leaved forest > coniferous forest > shrubs. The *M* of shrubs and coniferous forests showed a very significant increase with the increase of decomposition degree, while the *M* of broad-leaved forests showed a trend of first decreasing and then increasing with the increase of decomposition degree.

**Fig 3:**
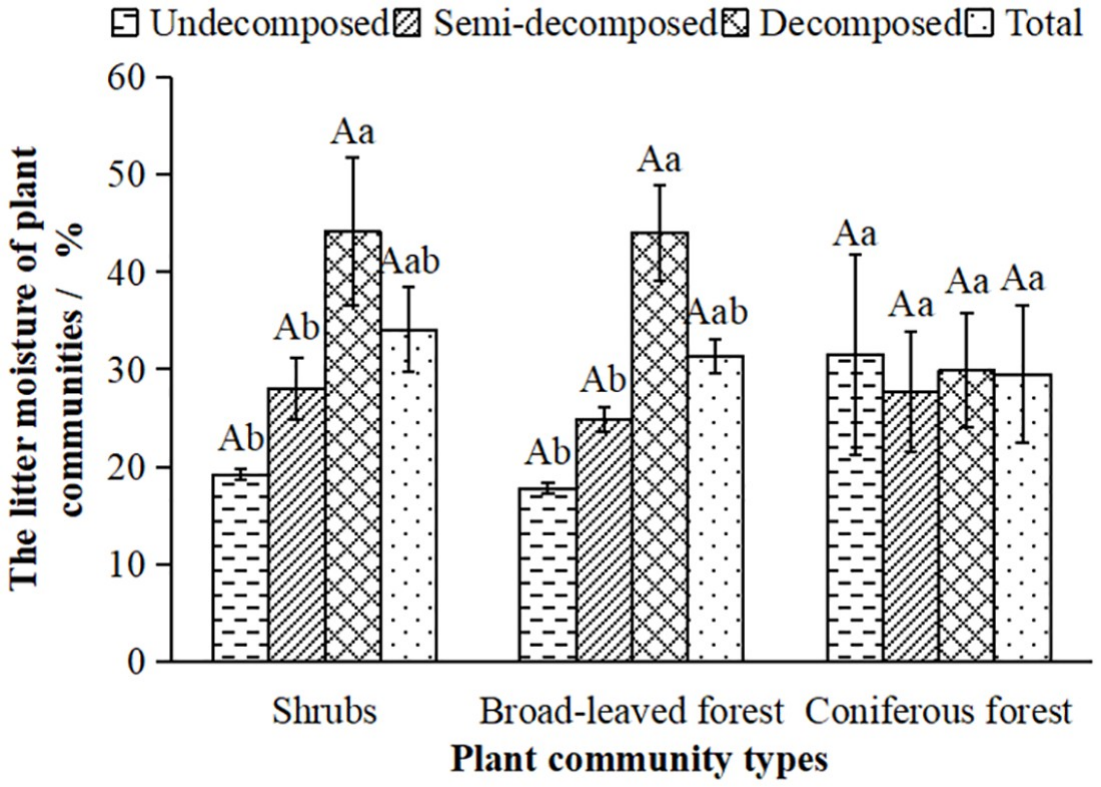
The moisture content(*R*_*n*_) of litter in the typical plant communities.

The moisture content(*R*_*n*_) of the litter layer is an important indicator that affects the hydrological regulation function of the plant community. The *R*_*n*_ directly affects the maximum interception capacity (rate) and the effective interception capacity (rate) of the litter. As shown in Fig 3, the *R*_*n*_ in the three typical plant communities was in the order: shrubs > broad-leaved forest > coniferous forest. Analysis of the *R*_*n*_ in different decomposition states showed that the *R*_*n*_ in shrubs and broad-leaved forests increased with the degree of decomposition. The *R*_*n*_ of coniferous forest decreased first and then increased.

**Fig 4:**
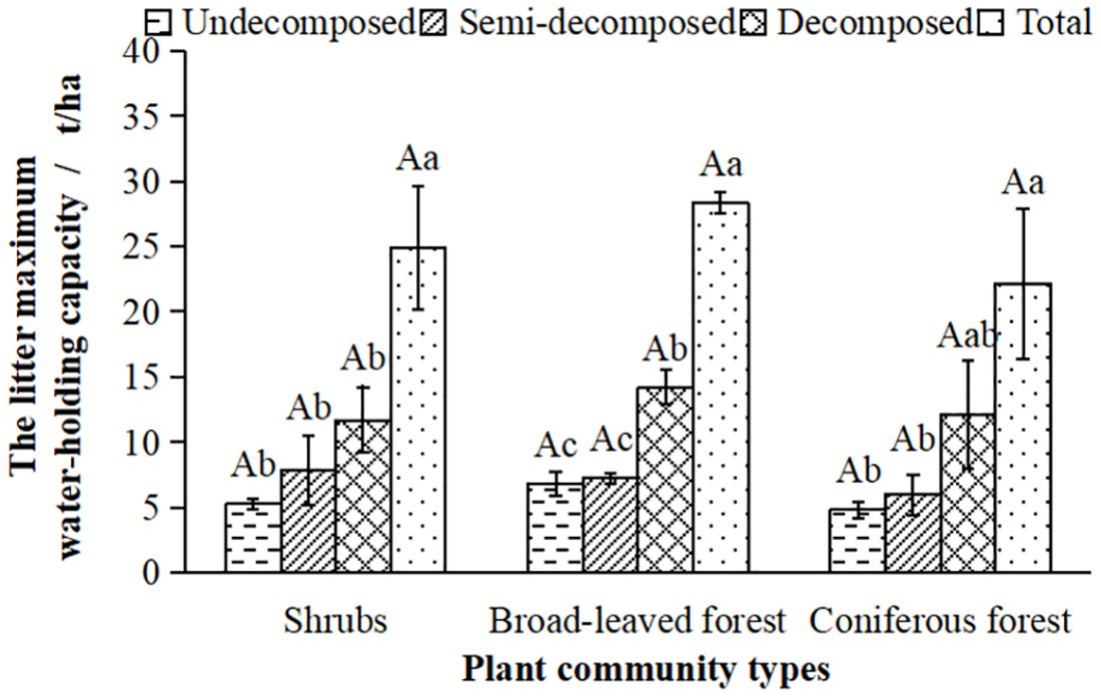
The maximum water-holding capacity(*M*_*wmax*_) of litter in the typical plant communities.

As shown in Fig 4, there was no significant difference in the maximum water-holding capacity(*M*_*wmax*_) of the three typical plant communities, and the changing trend of the *M*_*wmax*_ declined in order as broad-leaved forest > shrubs > coniferous forest. The *M*_*wmax*_ of the broad-leaved forest, shrubs, and coniferous forest showed a significant increase with the increase of decomposition degree, and there was no significant difference in the *M*_*wmax*_ in the same layers in these plant communities.

### 3.2 Analysis of hydrological functions of the litter layer

**Fig 5:**
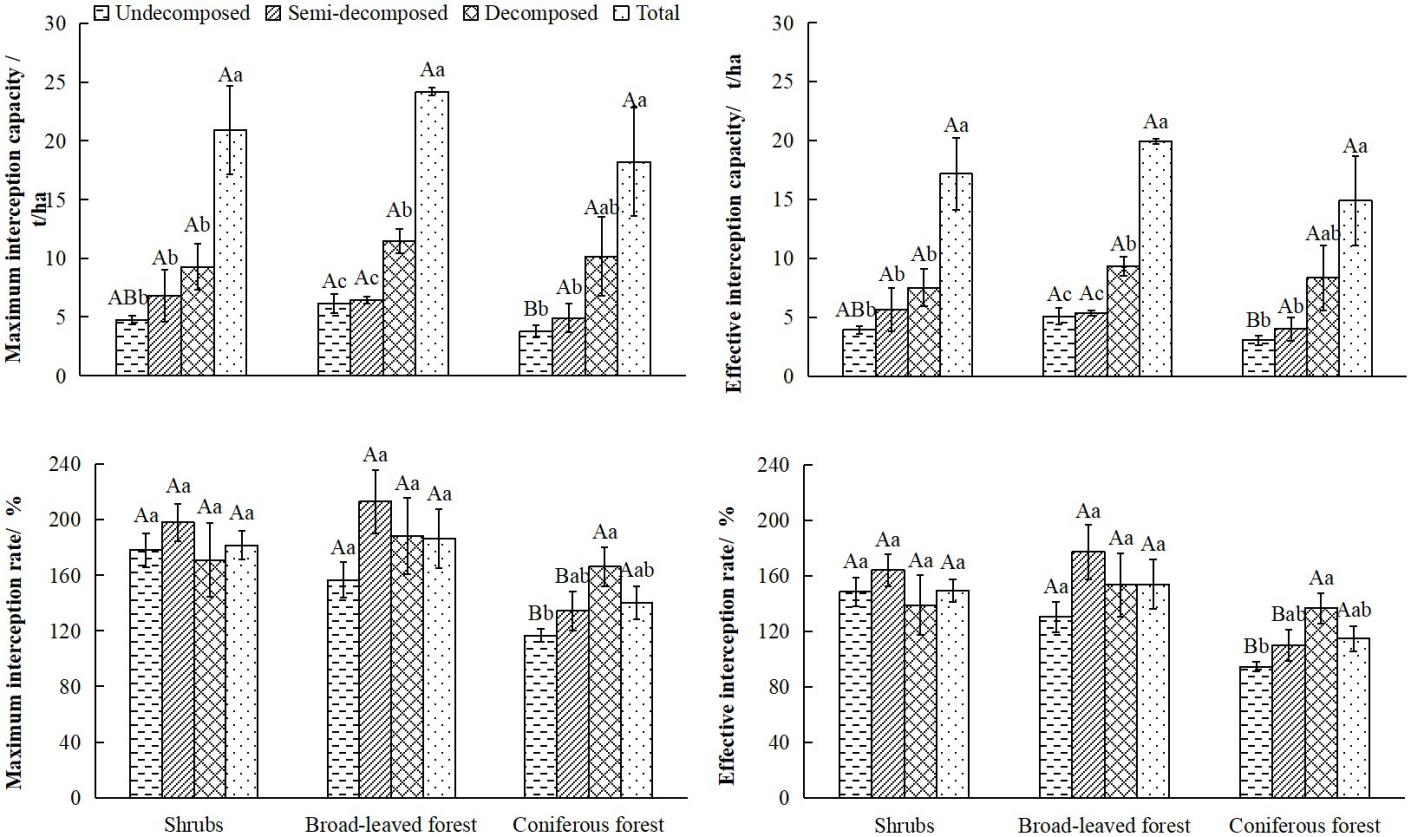
The hydrological characteristics of the litter in the three typical communities.

Generally, the maximum interception capacity(*M*_*lmax*_), the maximum interception rate (*Rl*_*max*_), the effective interception capacity(*M*_*sv*_), and the effective interception rate(*R*_*sv*_) are used to evaluate the ecological and hydrological functions of litter. Among them, the *M*_*lmax*_ and *Rl*_*max*_ can be used effectively to analyze the litter layer’s maximum water conservation capacity under natural conditions. The *M*_*sv*_ and *R*_*sv*_ are used to evaluate the interception and storage function of litter in response to rainfall. As shown in Fig 5, the *M*_*lmax*_, *Rl*_*max*_, *M*_*sv*_ and *R*_*sv*_ of the three typical plant communities presented the trend of broad-leaved forest > shrubs > coniferous forest. The *M*_*lmax*_ and the *M*_*sv*_ in these plant communities showed a significant increase with the increase of decomposition degree, while the change trends of the *Rl*_*max*_ and *R*_*sv*_ were different. The litter of shrubs and broad-leaved forests first increases and then decreases with the degree of decomposition, while the coniferous forest communities show a gradually increasing trend.

### 3.3 Analysis of " absorption -release" water process of litter

**Fig 6:**
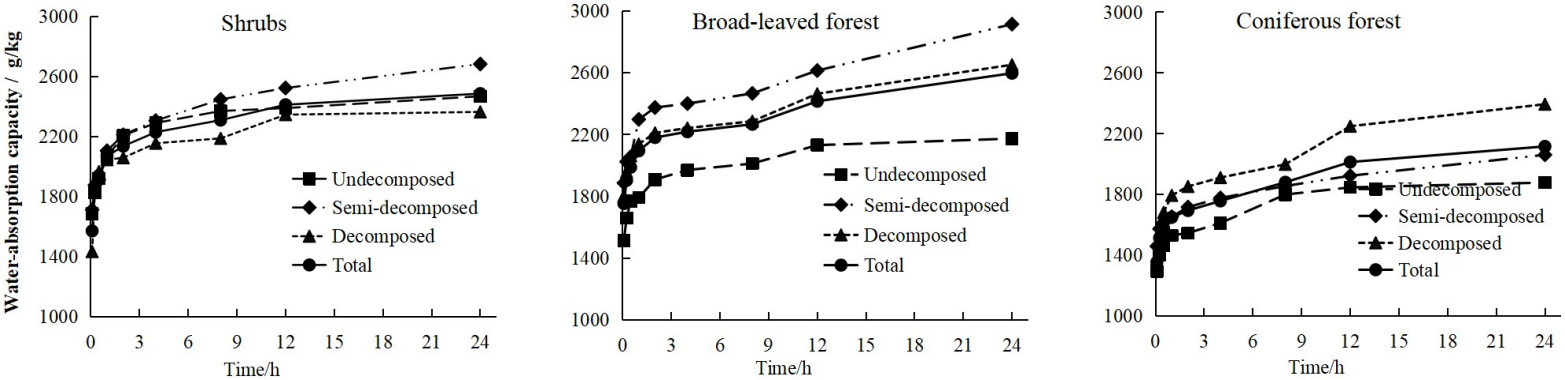
The water-absorption characteristics of litter in the different typical plant communities.

The litter layer realizes the functions of intercepting and regulating rainfall through the "water-absorption process" and the "water- release process," thereby exerting the functions of water conservation and rainfall regulation. It can be seen from Fig 6 that the water-absorption capacity(*Q*_*ct*_) and time of the litter layer of the three typical plant communities were all in logarithmic function models. The water-absorption process of the litter layer can be divided into three stages: the 0–15 min fast stage, the 15 min–2 h slow stage, and the 2–48 h gentle stage. The *Q*_*ct*_ showed a rapid increase trend in the first stage, and its cumulative *Q*_*ct*_ was in the range of 1402.13–2023.99 g/kg, reaching 67.02%–77.28% of the total water absorption of the litter layer. In the second stage, the *Q*_*ct*_ increased slowly, and the increase in the *Q*_*ct*_ was 142.99–379.51 g/kg, which accounted for 8.50%–15.38% of the total. In the third stage, the increase in the *Q*_*ct*_ slowly decreased, and the litter tended to be saturated. Correlation analysis between the litter water-absorption capacity(*Q*_*ct*_) and immersion time(*t*) of three typical plant communities (Table 1) revealed that the litter layer water-absorption capacity was significantly positively correlated with time (*P*<0.01), which is in line with a logarithmic function relationship (*Q*_*ct*_ = *b* + *a* ln *t*).

**Table 1:** The regression equation of the litter water-absorption capacity(*Q*_*ct*_) and immersion time(t).

Layer	Shrubs			Broad-leaved forest			Coniferous forest		
	Equation	R^2^	Sig	Equation	R^2^	Sig	Equation	R^2^	Sig
Undecomposed	*Q*_*ct*_ = 2052.33+144.31ln*t*	0.97	<0.01	*Q*_*ct*_ = 1814.79+114.10ln*t*	0.99	<0.01	*Q*_*ct*_ = 1535.46+105.91ln*t*	0.96	<0.01
Semi-decomposed	*Q*_*ct*_ = 2098.87+170.37ln*t*	0.99	<0.01	*Q*_*ct*_ = 2242.78+162.52ln*t*	0.93	<0.01	*Q*_*ct*_ = 1680.15+97.21ln*t*	0.96	<0.01
Decomposed	*Q*_*ct*_ = 1959.55+143.71ln*t*	0.91	<0.01	*Q*_*ct*_ = 2125.76+130.85ln*t*	0.94	<0.01	*Q*_*ct*_ = 1778.39+162.35ln*t*	0.95	<0.01
Total	*Q*_*ct*_ = 2022.66+152.45ln*t*	0.99	<0.01	*Q*_*ct*_ = 2079.56+134.09ln*t*	0.96	<0.01	*Q*_*ct*_ = 1655.26+125.87ln*t*	0.97	<0.01

**Fig 7:**
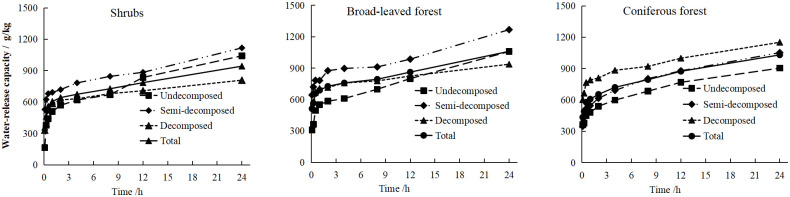
The water-releasing characteristics of the litter in the typical plant communities.

As shown in Fig 7, both the litter water-release capacity(*Q*_*st*_) and the immersion time(*t*) of the three typical plant communities showed power function models. The water-release process of litter can be divided into three stages, namely, the 0–1 h rapid stage, the 1–4 h slow stage, and the 4–48 h gentle stage. In the first stage, the amount of water released by the litter increased rapidly (481.69–791.29 g/kg), reaching 48.86%–75.45% of the total water released. During this stage, 20.62%–33.05% of the total water absorption of the litter was released.

In the second stage, the amount of water released by the litter slowly increased. The cumulative increase in the amount of water released was 44.97–145.04 g/kg, reaching 5.46%–13.77% of the total water released, which released 1.90%–7.04% of the total water-absorption of the litter. In the third stage, the amount of water released by the litter tended to be stable. Correlation analyses between time and the water-release capacity by the litter for the three typical plant communities were performed (Table 2). The water-release capacity (*Q*_*st*_) by litter was extremely significantly positively correlated with the immersion time(*t*), which conformed to a power function relationship (*Q*_*st*_ = *a t* ^*b*^).

**Table 2:** The regression equation of the litter water-releasing capacity(*Q*_*st*_) and time(*t*).

Layer	Shrubs			Broad-leaved forest			Coniferous forest		
	Equation	R^2^	Sig	Equation	R^2^	Sig	Equation	R^2^	Sig
Undecomposed	*Q*_*st*_ = 446.80*t*^0.27^	0.90	<0.01	*Q*_*st*_ = 509.68*t*^0.20^	0.95	<0.01	*Q*_*st*_ = 500.68*t*^0.16^	0.97	<0.01
Semi-decomposed	*Q*_*st*_ = 701.65*t*^0.11^	0.93	<0.01	*Q*_*st*_ = 811.15*t*^0.10^	0.89	<0.01	*Q*_*st*_ = 557.24*t*^0.19^	0.99	<0.01
Decomposed	*Q*_*st*_ = 522.20*t*^0.15^	0.92	<0.01	*Q*_*st*_ = 679.53*t*^0.09^	0.96	<0.01	*Q*_*st*_ = 780.94*t*^0.10^	0.97	<0.01
Total	*Q*_*st*_ = 566.79*t*^0.15^	0.96	<0.01	*Q*_*st*_ = 675.35*t*^0.11^	0.95	<0.01	*Q*_*st*_ = 612.07*t*^0.24^	0.98	<0.01

### 3.4 Analysis of water rate of the "absorption-release" of litter

**Fig 8:**
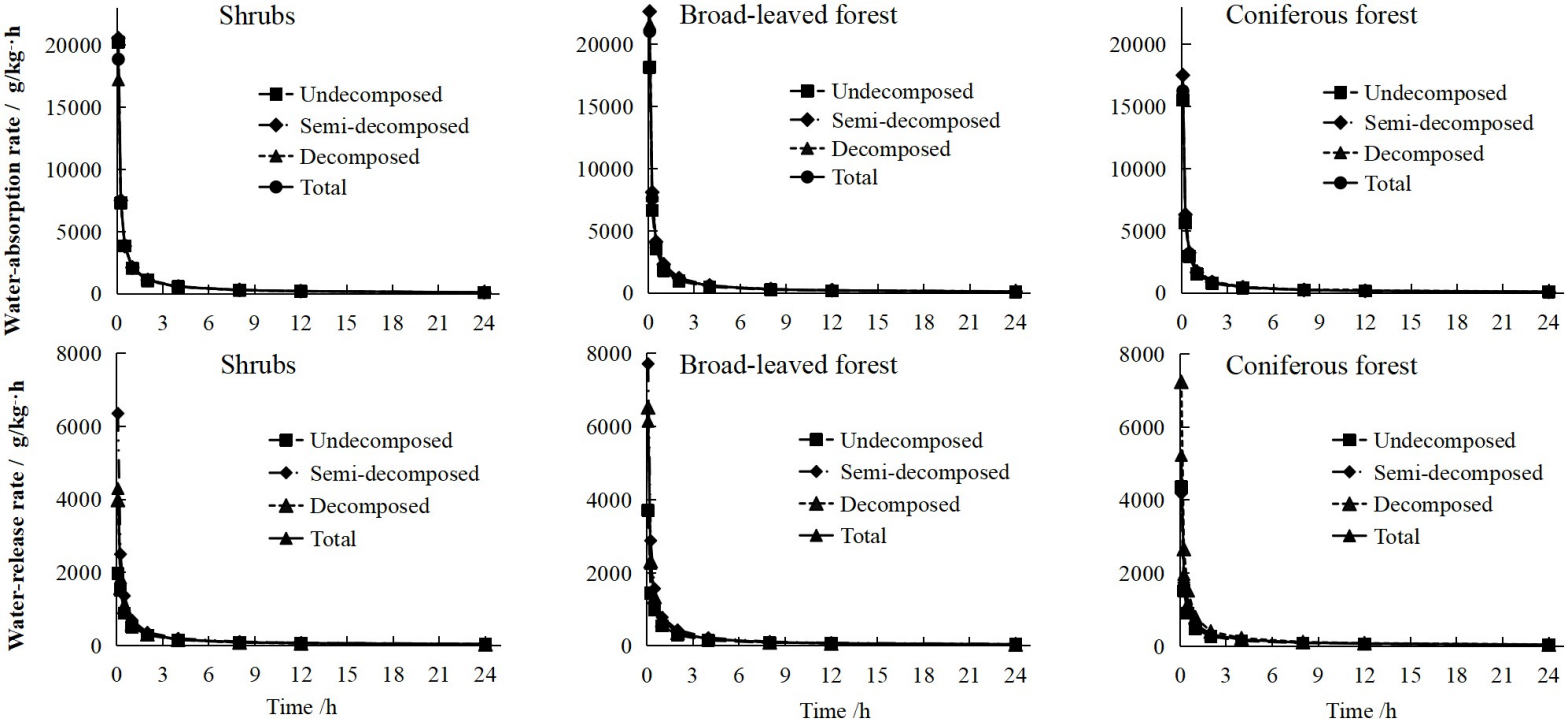
The litter water-absorption/release rate characteristics (g/kg·h).

The water-absorption rate (*v*_*c*_) of the litter layer directly reflects the rainfall interception speed of the litter layer and the capability of regulation of the regulation and storage function, while the water-release rate(*v*_*s*_) reflects the release speed of the litter layer to the intercepted rainfall and exerts the functions of delaying runoff and conserving water. The water absorption rate analysis of the three typical plant communities is shown in Fig 8. The changing trend of the litter water absorption rate can be divided into four stages. The first was the fast stage, the *v*_*c*_ was the highest within 0–5 min, at 15529.01–22634.43 g/kg·h. The second was the rapid water-absorption stage, and the *v*_*c*_ was greatly reduced to 2709.97–3922.87 g/kg·h within 5 min–2 h. The third stage was the slow water-absorption stage, during which the *v*_*c*_ slowly decreased to 313.87–454.17 g/kg·h within 2–8 h. The *v*_*c*_ in the fourth stage (8–48 h) was the lowest at only 116.06–169.63 g/kg·h.

The analysis of the water-release rate(*v*_*s*_) of three typical plant communities revealed that the changing trend of the *v*_*s*_ was consistent with the changing trend of the *v*_*c*_ and could be divided into four stages, but the *v*_*s*_ at different stages was much lower than the *v*_*c*_. In the first stage, the difference between the water "absorption-release" rate was one order of magnitude, and in the second to fourth stages, the difference was only 2.1 to 4.5 times. As shown in Table 3, the water absorption and release rates of litter were significantly negatively correlated with time (*P*<0.01), which was in line with the power function relationship (*v* = *a t* ^*-b*^).

**Table 3:** The regression equation of the litter water-absorption/release rate and time.

Layer		Shrubs			Broad-leaved forest			Coniferous forest		
		Equation	R^2^	Sig	Equation	R^2^	Sig	Equation	R^2^	Sig
Water-absorption	Undecomposed	*v*_*c*_ = 1509.50*t*^-0.91^	0.955	<0.01	*v*_*c*_ = 1925.61*t*^-0.94^	0.991	<0.01	*v*_*c*_ = 1790.88*t*^-0.94^	0.993	<0.01
	Semi-decomposed	*v*_*c*_ = 1743.95*t*^-0.92^	0.977	<0.01	*v*_*c*_ = 2188.78*t*^-0.95^	0.987	<0.01	*v*_*c*_ = 1946.28*t*^-0.92^	0.999	<0.01
	Decomposed	*v*_*c*_ = 1453.45*t*^-0.90^	0.983	<0.01	*v*_*c*_ = 2266.27*t*^-0.94^	0.997	<0.01	*v*_*c*_ = 2030.15*t*^-0.92^	0.996	<0.01
	Total	*v*_*c*_ = 1537.28*t*^-0.91^	0.982	<0.01	*v*_*c*_ = 2151.59*t*^-0.94^	0.994	<0.01	*v*_*c*_ = 1961.56*t*^-0.93^	0.999	<0.01
Water-release	Undecomposed	*v*_*s*_ = 369.76*t* ^-0.77^	0.87	<0.01	*v*_*s*_ = 576.61*t* ^-0.83^	0.98	<0.01	*v*_*s*_ = 432.38*t* ^-0.76^	0.87	<0.01
	Semi-decomposed	*v*_*s*_ = 609.08*t* ^-0.83^	0.95	<0.01	*v*_*s*_ = 627.95*t* ^-0.88^	0.92	<0.01	*v*_*s*_ = 705.67*t* ^-0.87^	0.99	<0.01
	Decomposed	*v*_*s*_ = 477.52*t* ^-0.91^	0.95	<0.01	*v*_*s*_ = 683.40*t* ^-0.90^	0.98	<0.01	*v*_*s*_ = 791.01*t* ^-0.91^	0.97	<0.01
	Total	*v*_*s*_ = 486.81*t* ^-0.85^	0.99	<0.01	*v*_*s*_ = 646.98*t* ^-0.88^	0.97	<0.01	*v*_*s*_ = 691.64*t* ^-0.88^	0.98	<0.01

## 4 Discussion

The water-holding capacity (moisture content, maximum water-holding capacity, maximum water-holding rate, etc.) of the litter layer is one of the important indicators reflecting the hydrological effect of the forest. Its reserves directly affect the hydrological functions of the plant community. There are many factors that influence the number of litter reserves in plant communities, including the community type, the season, the plant density, the community age, the species composition, the topography, etc., but the primary factor is the type of plant communities [43–45]. Bai et al.’s [46] study of the litter reserves in the coniferous and broad-leaved forests showed that the litter reserves were closely related to the plant community type. This study showed that there was no significant difference in the total litter reserves(*M*) of the three typical plant communities in the area of Karst peak-cluster depressions. However, as the degree of decomposition increased, the *M* of broad-leaved forests showed a trend of first decreasing and then increasing. The *M* of typical plant communities was significantly lower than the results of the study by Tu *et al*. [1] on the *M* (23.20–39.11 t/ha) of forest ecosystems in the Dahuofang Reservoir watershed. The reason for this may be that the differences between different plant communities are related both to species composition and the growth characteristics of the dominant species, and to the environment in which each plant community is located. The analysis results of the *M* (10.14–25.07 t/ha) of five typical forest types in Zhejiang Province found by Sun *et al*. [47] were relatively close to the values found in this study. The changing trend of litter reserves in different decomposition layers was similar to that found by Zhang *et al*. [3] in *Rhododendron sibiricum* forest in a nature reserve. The large surface area of bracken litter in Pitman’s study resulted in higher litter reserves and a relatively high water uptake rate compared to the litter from other species [48]. This is similar to the results of this study, where the bracken is a broad-leaved plant and the broad-leaved plant has a larger leaf area and higher total litter reserves than shrubs and conifers. The wider the fronds or the larger the total area, the larger the reservoir and the greater the water storage capacity, and the higher the litter reserves [49]. Putuhena et al. [50] showed that the higher the maximum water-holding capacity of the litter, the greater the regulation of precipitation. Bulcock et al. [51] studied the water-holding capacity of litter in three different forest types in South Africa, with broad-leaved plants having the best water-holding capacity. However, the water-holding capacity of coniferous and broad-leaved forest litter was found to be almost identical in the eastern foothills of the Alps by Katalin et al. [44], which differs from the results of the present study. Differences in the water-holding capacity of the litter may be related to factors such as the leaf structure and physicochemical properties of the litter [52].

The interception function of the litter in plant communities depends on litter reserves and water-holding properties [45]. The moisture content(*R*_*n*_) of the litter layer of the three typical Karst peak-cluster depressions plant communities was in the order of shrubs > broad-leaved forest > coniferous forest. The changing trend of the maximum water-holding capacity(*M*_*wmax*_) of the plant communities was broad-leaved forest >shrubs >coniferous forest. The changing trends of the interception rate(*Rl*_*max*_), effective interception capacity(*M*_*sv*_), and effective interception rate(*R*_*sv*_) were consistent with the changing trend of the maximum interception capacity (*M*_*lmax*_). This result shows that the litter layer of the broad-leaved forest community has the strongest ability to regulate precipitation, and its hydrological service function is the best. The higher the degree of litter decomposition, the stronger the interception function. This was consistent with the results obtained by Yang and Zhang *et al*. [6] on the water conservation capacity of the litter layer of three mixed forest types in the area along the dam, in which the maximum water-holding capacity and effective interception capacity of the litter layer were broad-leaved mixed forest > mixed coniferous broad-leaved forest > coniferous mixed forest. In Helvey’s study [53], it was also shown that the interception function of broad-leaved forests is optimal. The decomposition of litter differs between plant community types, generally, broad-leaved forests > mixed coniferous forests > coniferous forests. The results of this study also follow the general pattern. The decomposition of litter is mainly influenced by environmental factors such as temperature and humidity, as well as by biological activity in the soil [54]. The age and composition of the forest, topographic factors, cultural treatments, and seasonal variations also have a significant impact on the ability of litter interception [55]. In general broad-leaved litter decomposes faster than coniferous litter, due to the higher average annual temperature at the site of decomposition than coniferous litter, in addition to factors such as the high nutrient content of broad-leaved litter and the lower carbon fraction [56]. Related analyses also suggest that the decomposition of leaf litter in China is controlled by climate and litter properties, as has been reported in large-scale studies from other regions (e.g. North America, South America and Europe) [57, 58]. The results on the decomposition degree and interception function were consistent with the results of Xuan *et al*. [59] on the hydrological characteristics of the litter layer of four forest densities of *Pinus tabulaeformis* plantations in the northern Hebei mountainous area, in which the interception capacity of litter in the semi-decomposed layer was greater than that in the undecomposed layer. Luo *et al*. [60] obtained similar results in the study of the litter layer water-holding characteristics of different vegetation types in a small watershed in northwestern Hunan. The maximum water-holding rate of different vegetation types was the semi-decomposed layer (220. 64%) > the undecomposed layer (216. 15%), indicating that the water-holding capacity of litter in the semi-decomposed layer was greater than that of the undecomposed layer. As part of the semi-decomposed layer has decomposed or is in the process of decomposing, the structure of the litter is looser and the voids gradually increase, allowing more water to be intercepted, so the water-holding capacity is at its best in the semi-decomposed layer of the litter [45].

The litter water-absorption capacity(*Q*_*ct*_) of three typical Karst peak-cluster depressions plant communities showed logarithmic function models over time(*t*). The water-absorption process of litter can be divided into three stages. In the first stage, the cumulative *Q*_*ct*_ of litter accounted for 67.02%–77.28% of the total *Q*_*ct*_ of litter. This result is consistent with the research results of Feng *et al*. [61] on the water conservation function of litter in different forests in the Guansi River Basin in Mianyang. The relationship between water-absorption capacity and immersion time of litter satisfies the equation *Q = a*ln *t+b*. The trend of litter water-release capacity(*Q*_*st*_) is the same as that of water-absorption capacity(*Q*_*ct*_). In the first stage, the cumulative *Q*_*st*_ of litter accounted for 48.86%–75.45% of the total *Q*_*st*_, and only 20.62%–33.05% of the total water-absorption capacity(*Q*_*ct*_) of litter was released. The *Q*_*st*_ by litter is significantly positively correlated with time (*P*<0.01), which conforms to a power function relationship (*Q*_*st*_ = *a t* ^*b*^). This result is somewhat different from that of Zhang *et al*. [62] on the water-holding characteristics of plantation litter in the terraced area of Ziquejie in Hunan Province, and they concluded that the most suitable model for litter release and time was *Q* = *b + a*ln *t*. Liu e*t al*. [63] studied the hydrological characteristics of larch forest litter in the southern mountainous area of Ningxia and concluded that the most appropriate model for the amount of water released by the litter and time was Q = a *t* ^*-b*^, and they used litter moisture content for analysis, while this study used the litter accumulated water release for analysis.

The rate of water absorption by litter can reflect the magnitude of precipitation and interception capacity in the forest, and the stronger the interception capacity can effectively inhibit soil water evaporation and delay surface runoff [5, 6]. The litter water absorption and release rates of the three typical Karst peak-cluster depressions plant communities showed a four-stage change with time. However, the water release rate(*v*_*s*_) at different stages was lower than the water absorption rate(*v*_*c*_). The *v*_*c*_ and *v*_*s*_ in the first stage differed by an order of magnitude, and in the second to fourth stages differed by 2.1–4.5 times. The *v*_*c*_ and *v*_*s*_ of litter were significantly negatively correlated with time (*P*<0.01), which conformed to the power function relationship (*v* = *a t* ^*-b*^). This research result was somewhat different from the research results of Peng *et al*. [64] on the water-holding capacity of litter in the water conservation forest at the source of the Hunhe River. They concluded that the water absorption rate of litter reaches its peak during 0–1 h, and the water absorption rate dropped rapidly in 1–4 h and then stabilized, which was consistent with the research results of Zhang *et al*. [3] on the water-holding process of the litter layer of the Rhododendron forest in Baili Rhododendron Nature Reserve, Guizhou. The reasons for the differences may be related to the structure and chemical properties of the litter of different forest tree species, etc. The water absorption (release) equation of litter is similar to the results of Zhou *et al*. [65] on the water-holding capacity of typical forests in the Chongqing Jinyun Mountain National Nature Reserve. The results reveal that for the water-absorption rate and water release rate, the optimal model of speed and time is *V = a t*^*b*^. Zhou *et al*. [66] obtained the same results in the study of the hydrological and ecological functions of the litter layer of *Robinia pseudoacacia* forest with different densities in the loess area of western Shanxi. The total water absorption rate of the litter of the three plant communities reached the highest in the broad-leaved forest, further indicating that the broad-leaved forest has the strongest water storage.

## 5 Conclusion

In this study, the litter reserves and the hydrological functions and process characteristics of three typical Karst plant communities were analyzed around FAST. The results show that the litter layer of the broad-leaved forest plant community has the strongest function of intercepting and regulating precipitation, and its hydrological service function is the best. The higher the degree of litter decomposition, the stronger the interception function. The litter water-absorption capacity (*Q*_*ct*_) of the three typical plant communities corresponded to a logarithmic function with time (*t*). The litter water-release capacity (*Q*_*st*_) corresponded to a power function with time (*t*). The water absorption and release rates of litter (*v*_*c*_ and *v*_*s*_) corresponded to a power function with time (*t*).
